# Management of Multidrug-Resistant Acinetobacter Infection Following Open Reduction and Internal Fixation of a Right Acetabular Fracture: A Case Report

**DOI:** 10.7759/cureus.83125

**Published:** 2025-04-28

**Authors:** Rajeev P Nagassar, Darian Singh, Raakesh Goalan

**Affiliations:** 1 Department of Microbiology, Sangre Grande Hospital, Sangre Grande, TTO; 2 Department of Orthopedics, Sangre Grande Hospital, Sangre Grande, TTO; 3 Department of Surgery, Sangre Grande Hospital, Sangre Grande, TTO

**Keywords:** acinetobacter, amikacin, complex surgical infection, local antibiotic therapy, multidrug resistant infections, open reduction and internal fixation infection, orthopedics, surgical infections, systemic antibiotic therapy, trauma

## Abstract

Postoperative infections with multidrug-resistant (MDR) *Acinetobacter baumannii* present significant challenges in orthopedic surgery. This case report highlights the management and successful treatment of an MDR *Acinetobacter* infection following open reduction and internal fixation (ORIF) of a right acetabulum fracture. A 51-year-old male presented with a comminuted right acetabular fracture following a motor vehicle accident. Initial ORIF was complicated by intraoperative blood loss, necessitating early termination of the procedure. The patient subsequently developed a deep surgical site infection caused by MDR *A. baumannii*, resistant to multiple antibiotic classes. This case underscores the importance of culture-guided antibiotic selection, a role for the use of local antibiotic therapy. It also highlights therapeutic challenges in managing MDR infections in orthopedic surgery and the need for collaboration with the microbiology team.

## Introduction

Surgical site infections (SSIs) following orthopedic procedures pose significant risks, especially when caused by multidrug-resistant organisms (MDROs) [1]. *Acinetobacter baumannii* is an opportunistic pathogen known for its resistance to multiple antibiotics, complicating treatment options in human medical practice [2]. This report discusses an MDR *Acinetobacter *infection following open reduction and internal fixation (ORIF) of an acetabular fracture, focusing on therapeutic strategies and a patient’s outcome. We also highlight interdisciplinary collaboration for a successful patient outcome.

## Case presentation

A 51-year-old male with a history of diabetes mellitus presented with a right acetabular fracture after a motor vehicle accident as part of a polytraumatic injury. Initial management at another institution included temporary hip stabilization using skeletal traction with a Steinmann pin in the proximal tibia and an immediate reduction and fixation of an ipsilateral midfoot injury. On admission, laboratory investigations revealed a mild anemia with mildly elevated white blood count (14.26 x10^9^/L; upper limit 12x10^9^/L). Other blood parameters were within normal ranges (see Table 1).

**Table 1:** Serial Blood Investigations During the Patient's Hospital Stay WBC: White blood cells; Hb: Hemoglobin; BUN: Blood urea nitrogen; CRP: C-reactive protein

Date	WBC (x10^3/uL) (Normal Range: 3.70-10.10)	Hb (g/dL) (Normal Range: 12.90-15.90)	Platelets (x10^3/uL) (Normal Range: 155.00-366.00)	Sodium (mmol/L) (Normal Range: 136.00-145.00)	Potassium (mmol/L) (Normal Range: 3.50-5.10)	BUN (mg/dL) (Normal Range: 6.00-20.00)	Creatinine (mg/dL) (Normal Range: 0.50-1.20)	CRP (mg/L) (Normal Range: 0.10-5.00)
7/20/2023	10.16	11.5	475.5	135	4.58	14	0.5	-
8/1/2023	8.58	11.04	547.7	-	-	-	-	168.67
8/4/2023	10.66	10.1	452.4	-	-	-	-	263.66
8/14/2023	7.93	10.25	460.4	-	-	-	-	47.56
8/31/2023	6.52	10.38	382.4	-	-	-	-	44.54

**Figure 1:**
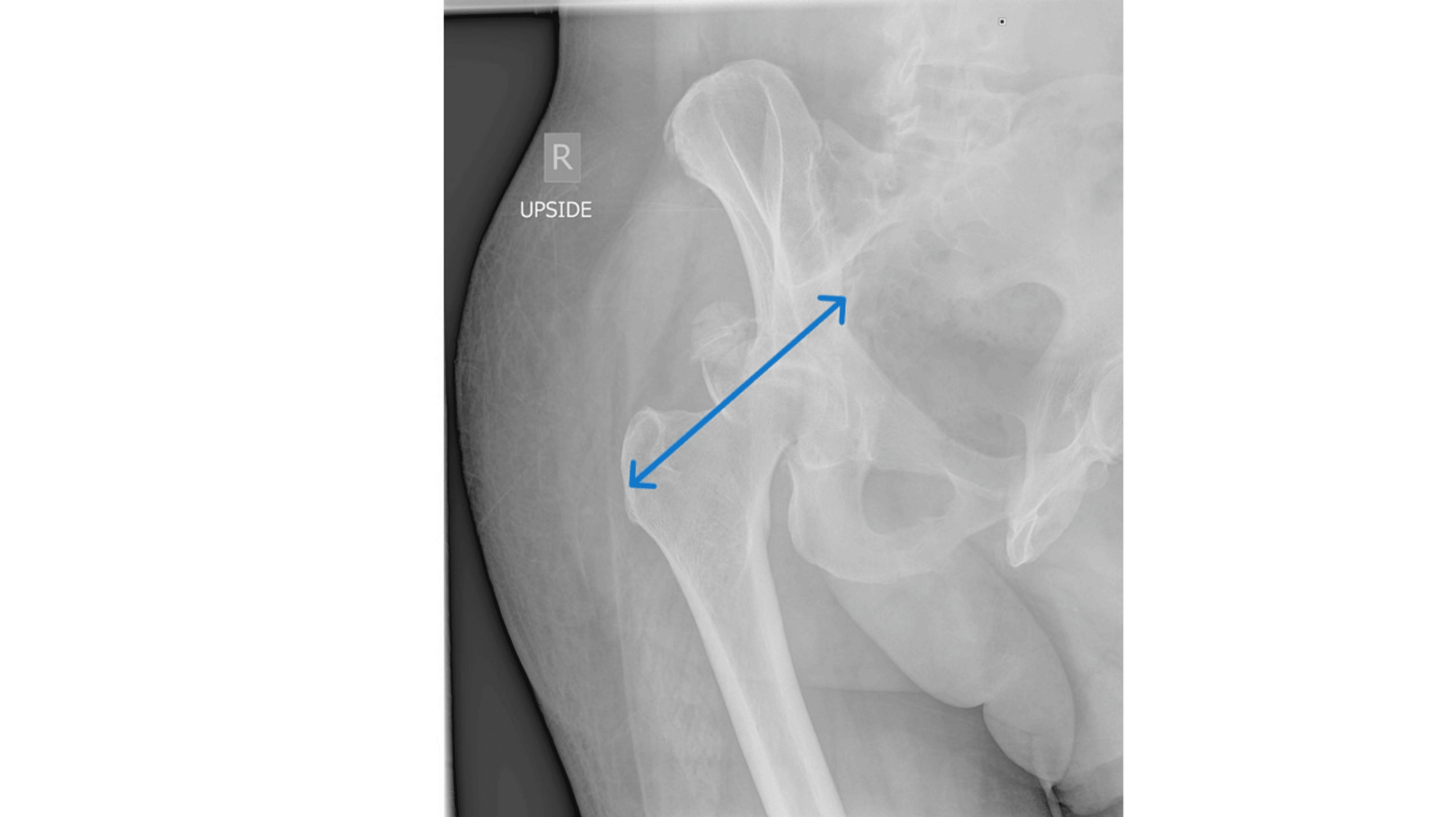
X-ray Image - Judet View Preoperative Judet view of the right hip showing the injury immediately following the accident. The area of interest is highlighted by the blue arrow.

**Figure 2:**
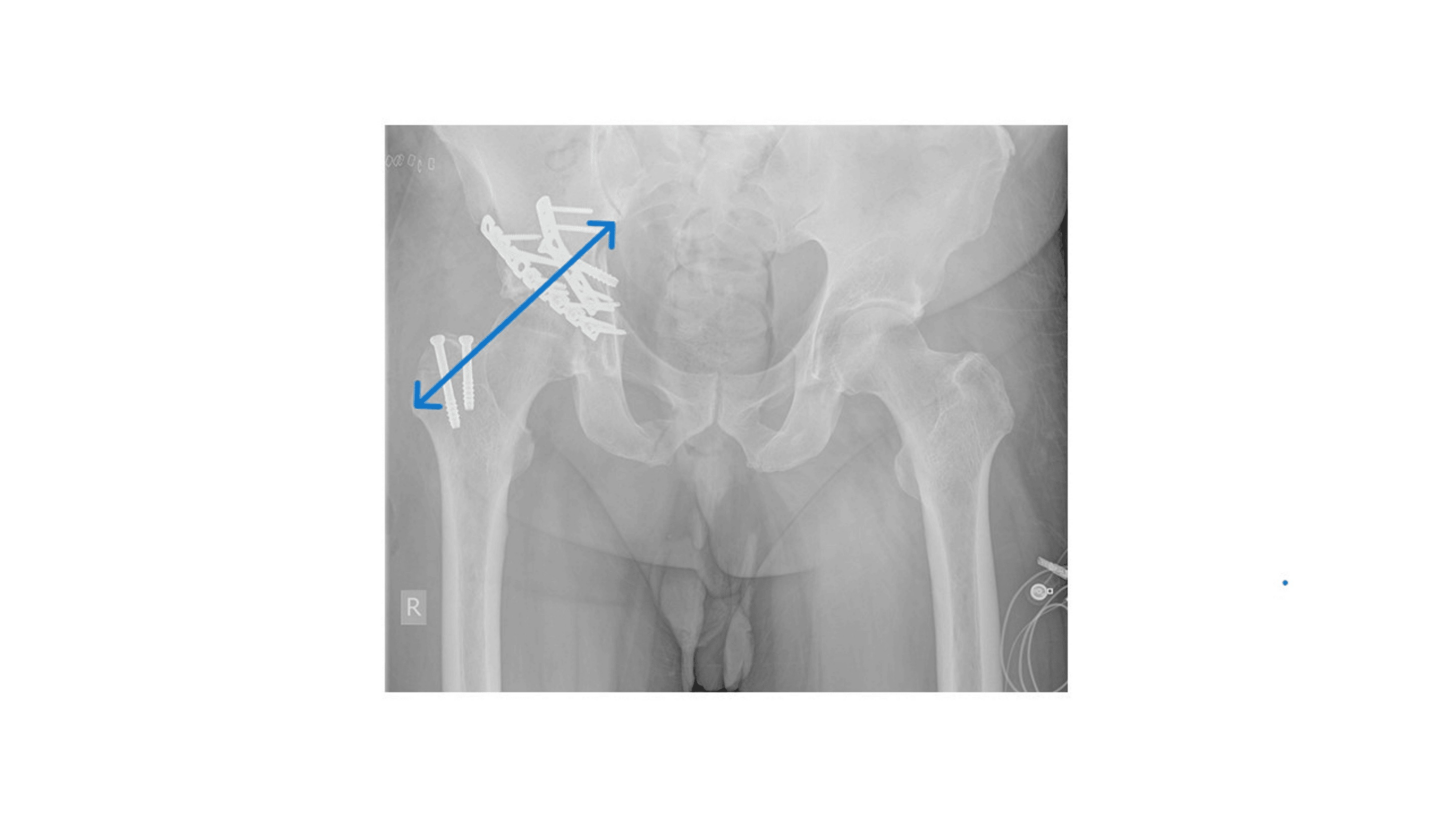
X-ray Image - AP View With Implants Postoperative fixation of the pelvis. The above anterior-posterior (AP) view shows fixation devices in situ (plates and screws) immediately after surgery. The area of interest is highlighted by the blue arrow.

An attempt was made to surgically fix the acetabulum during the week of admission, but the patient became unstable intra-operatively due to blood loss, and the procedure was abandoned. One week later, after an appropriate resuscitation, fixation was performed without complication (see Figures 1-2).

**Figure 3:**
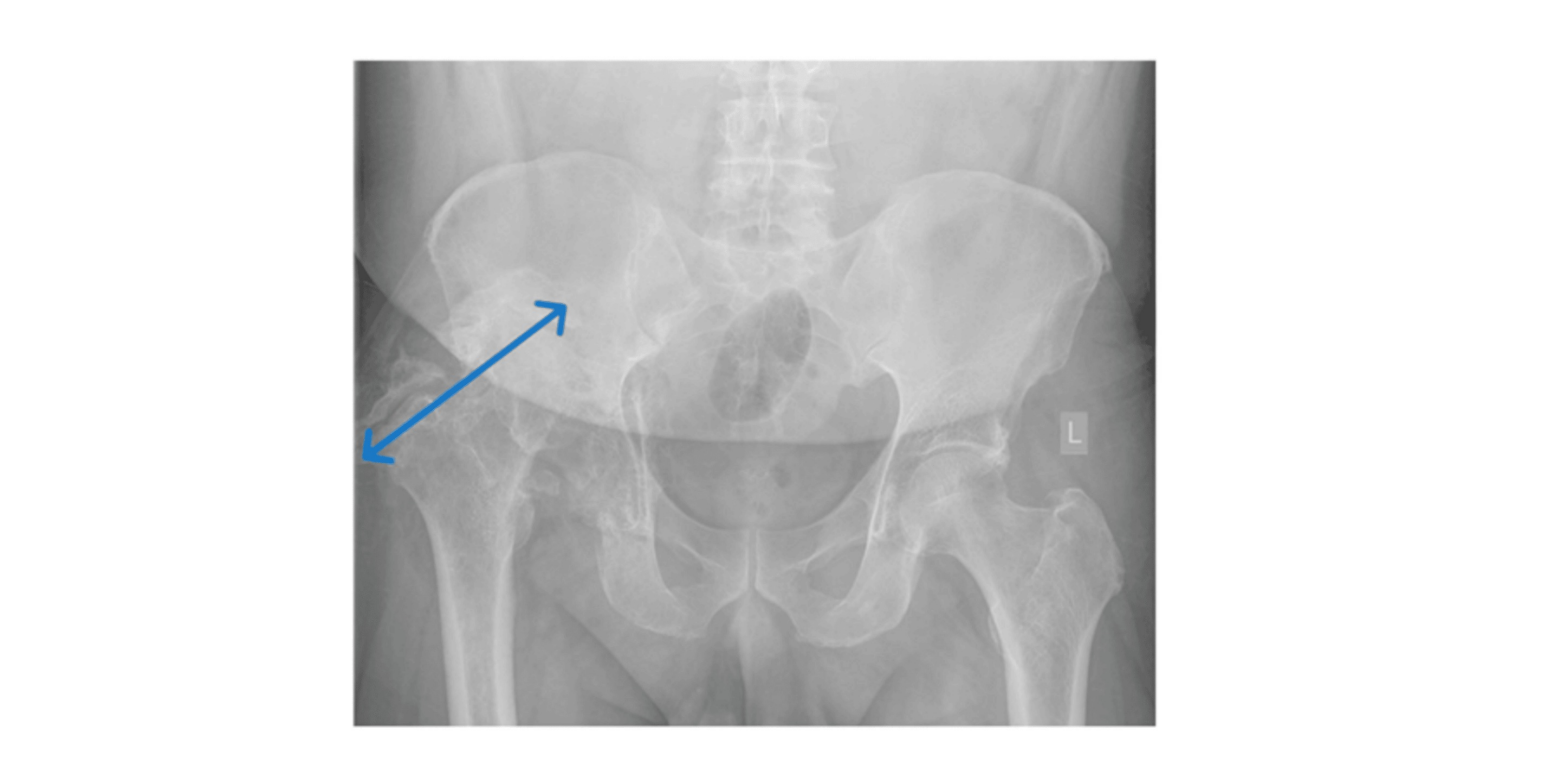
X-ray Image - AP View Post Removal Anterior-posterior (AP) view of the pelvis after removal of the metalwork due to infection. The area of interest is highlighted by the blue arrow.

On postoperative day 11, the patient presented with signs of wound infection. This included increased pain at the site and a cloudy fluid draining from the wound drain. Laboratory results showed an elevated CRP (168.67 mg/L) (normal range <10). See Table 1 for further blood investigations and the corresponding values. This prompted empirical broad-spectrum antibiotic coverage with piperacillin-tazobactam. The patient later had irrigation and debridement in theater with retained implants and then again, two weeks later, for removal of all metal implants when the infection failed to resolve. See Figure 3 for treatment interventions and the corresponding CRP results.

The initial wound swabs, taken on the ward, did not yield growth. Tissue cultures later identified MDR *A. baumannii*, resistant to all standard antibiotics except amikacin. The organism was resistant to the carbapenems, including meropenem and imipenem; however, they were incorporated into therapy for synergy and maximizing pharmacokinetic and pharmacodynamic parameters by infusion and using a high dosage over a prolonged period, three times daily (see Table 2).

**Table 2:** Antibiotics Used, Routes of Administration, and Duration of Treatment * The patient was discharged to go home. The patient received all doses of the antibiotics.

Antibiotics	Dose	Route of Administration	Frequency of Administration	Duration in days
Cefuroxime	1.5 g	Intravenous	3 times daily	10 days
Tazobactam-Piperacillin	4.5 g	Intravenous	4 times daily	2 days
Meropenem	1 g	Intravenous	3 times daily	1 day
Vancomycin	1 g	Intravenous	2 times daily	1 day
Tigecycline	100 mg	Intravenous	Immediately, once	1 day
Tigecycline	50 mg	locally	Immediately, once	1 day
Tigecycline	50mg	intravenously	2 times daily	2 days
Tigecycline	50mg in 100 mL of normal saline	Locally	2 times daily	2 days
Amikacin	400 mg	Intravenous	3 times daily	30 days
Amikacin	400 mg in 100 mL of normal saline	Locally	3 times daily	30 days
Meropenem	2 g	Intravenously	3 times daily	30 days
Trimethoprim-Sulfamethoxazole	960 mg	Orally	2 times a day	13 days
Linezolid	600 mg	Intravenously	2 times a day	3 days
Linezolid	600 mg	Orally*	2 times a day	5 days*

Dual antibiotic therapy was then started with amikacin as well as meropenem since imipenem was not available in the hospital pharmacy. The amikacin was also instilled directly into the wound via a surgical drain left in situ after the last explant procedure. After 14 days of therapy, inflammatory markers improved (see Tables 1-2 for a summary). The Medical Microbiologist was involved at all times in management in a multidisciplinary approach. The Becton Dickinson (BD) Phoenix Automated Microbiology System (BD Diagnostic Systems, Sparks, MD, USA) was used for identification and susceptibility testing, following the latest Clinical and Laboratory Standards Institute (CLSI) M100 guidelines.

The patient was ultimately discharged on oral linezolid with close outpatient follow-up (see Table 2). The microbiology data did not show any Gram-positive organisms. The patient was given oral linezolid to cover normal flora, and because he was still having pain, although decreased, and his CRP was still elevated despite being on over a month of culture-directed antibiotics. This was a deep wound, and we could not reach the site for further samples, as it had healed. Following the maintenance of low inflammatory markers and lack of clinical signs of infection, the patient eventually had a complex primary right total hip replacement done in another country and is undergoing rehabilitation to optimize his functional mobility.

## Discussion

*A. baumanii,* first described in 1911 and later renamed in 1986, is a Gram-negative coccobacillus, which, unlike other soil microbiota, is primarily a nosocomial pathogen [3]. It forms one of the ESKAPE organisms (*Enterococcus faecium*, *Staphylococcus aureus*, *Klebsiella pneumoniae*, *A. baumannii*, *Pseudomonas aeruginosa*, and *Enterobacter* species), which are known both for causing nosocomial infections and having a predilection for multidrug resistance [4]. The patient, being a diabetic with a long hospital stay and an indwelling catheter, provided a fertile milieu of risk factors for this opportunistic organism [5,6].

*A. baumanii *has been documented in the involvement of both colonizing and causing infections, with the respiratory, integumentary, central nervous, urinary, and hematological systems being involved [7,8]. Its involvement in trauma and orthopedics has been mainly described prior to post-traumatic injuries, often associated with the battlefield; however, other high-energy mechanisms may now present a similar breeding ground for affecting deep tissues surrounding trauma implants or osteomyelitis [9]. The patient was a victim of a high-energy injury, and this may have been one additional risk factor for succumbing to this pathogen. Use of multiple broad-spectrum antibiotics on the patient also represents a risk factor for the acquisition of *A. baumanii* infection [10].

However, the literature is scarce for the involvement of orthopedic wound sites. Even when trauma patients treated with metal implants are involved, *A. baumanii *is isolated from blood or urine cultures but not the surgical wounds [10]. This adds to the novelty of this case presentation. Only one study looked specifically at the involvement of arthroplasty and other trauma implants with respect to SSIs and identified MDR *A. baumanii* as the most common Gram-negative cause of infections [11]. As per the formula for rational empirical antibiotic therapy (FRAT), implant-related infections require combinations of amikacin, imipenem, and ciprofloxacin [12]. Our patient had sensitivity eventually to amikacin only, and we did not have access to imipenem, so the amikacin/meropenem combination was employed as highlighted in Table 2.

Given the deep-tissue nature of the SSI and the inability to delineate bony involvement on radiographic studies, an attempt was made to increase antibiotic concentration at the levels of both bone and deep soft tissue. Direct installation of antibiotics into the affected space was performed in an attempt to increase the concentration available to the infected tissue, despite the evidence being weak or non-existent for the same [13]. There are no studies easily available that discuss osteomyelitis secondary to *A. baumanii *in a clinical scenario, although a recent murine study does suggest that such a clinical entity would indeed hamper bony healing [14]. Notably, a review article has highlighted the emergence of *A. baumannii *as a successful pathogen and not just an environmental contaminant [15]. In terms of the use of local antibiotics, this has also been seen in urology, and we also see a place for it in orthopedics [16]. Various case series reports, literature reviews, and systematic reviews have supported the use of local antibiotic therapy in deep-seated joint infections [17-19]. This includes the use of catheters [17]. This is similar to our case presentation. These studies have supported the use of vancomycin, which is similar to our suggestion of linezolid. Importantly, the Medical Microbiologist played an important role in the management of the infection through surveillance (identification of the resistant infection), infection prevention and control, and antimicrobial stewardship [20,21].

Limitations

This being a case study, there are limitations such as generalizability and a lack of more empirical data. This case study, however, can motivate persons and serve as a catalyst to do more studies, including more scientifically rigorous studies, including clinical trials.

## Conclusions

MDR *Acinetobacter* infections in orthopedic trauma are rare but can be life-threatening. The failure of empirical therapy highlights the necessity of aggressive debridement, early culture-directed treatment, and the consideration of local antibiotic treatment, as has been seen in urology. Previous literature has shown similar cases requiring extensive hospital stays and multiple antibiotic adjustments. This case underscores the importance of early identification and targeted antibiotic therapy, the role of surgical debridement in infection control, and the necessity for multidisciplinary collaboration in managing complex infections.
